# Mealtime media use and cardiometabolic risk in children

**DOI:** 10.1017/S1368980020003821

**Published:** 2022-03

**Authors:** Joseph Jamnik, Charles Keown-Stoneman, Karen M Eny, Jonathon L Maguire, Catherine S Birken, for the TARGet Kids! Collaboration

**Affiliations:** 1 Child Health Evaluative Sciences, SickKids Research Institute, Toronto, Ontario, Canada; 2 The Applied Health Research Centre of the Li Ka Shing Knowledge Institute of St. Michael’s Hospital, Toronto, Ontario, Canada; 3 Division of Biostatistics, Dalla Lana School of Public Health, University of Toronto, Toronto, Ontario, Canada; 4 Nutrigenomix Inc., Toronto, Ontario, Canada; 5 Department of Pediatrics, Faculty of Medicine, University of Toronto, Toronto, Ontario, Canada; 6 Department of Pediatrics, St. Michael’s Hospital, Toronto, Ontario, Canada; 7 Institute of Health Policy, Management and Evaluation, Dalla Lana School of Public Health, University of Toronto, Toronto, Ontario, Canada; 8 Department of Nutritional Sciences, Faculty of Medicine, University of Toronto, Toronto, Ontario, Canada

**Keywords:** Mealtime media use, Cardiometabolic risk, Lipids, Children

## Abstract

**Objectives:**

To examine the association between mealtime media use and non-HDL-cholesterol as well as other markers of cardiometabolic risk (CMR) in children.

**Design:**

A repeated measures study design was used to examine the association between mealtime media use and CMR outcomes. Multivariable linear regression with generalised estimating equations was used to examine the association between mealtime media use and CMR outcomes. Analyses were stratified *a priori* by age groups (1–4 and 5–13 years).

**Setting:**

The TARGet Kids! Practice-based research network in Toronto, Canada.

**Participants:**

2117 children aged 1–13 years were included in the analysis.

**Results:**

After adjusting for covariates, there was no evidence that total mealtime media use was associated with non-HDL-cholesterol in 1–4 year olds (*P* = 0·10) or 5–13 year olds (*P* = 0·29). Each additional meal with media per week was associated with decreased HDL-cholesterol in 5–13 year olds (−0·006 mmol/l; 95 % CI −0·009, −0·002; *P* = 0·003) and log-TAG in 1–4 year olds (*β* = −0·004; 95 % CI −0·008, −0·00009; *P* = 0·04). Media use during breakfast was associated with decreased HDL-cholesterol in 5–13 year olds (−0·012 mmol/l; 95 % CI −0·02, −0·004; *P* = 0·002), while media during lunch was associated with decreased log-TAG (−0·01 mmol/l; 95 % CI −0·03, −0·002; *P* = 0·03) in children aged 1–4 years. Total mealtime media use was not associated with total cholesterol, glucose or insulin in either age group.

**Conclusions:**

Mealtime media use may be associated with unfavourable lipid profiles through effects on HDL-cholesterol in school-aged children but likely not in pre-schoolers.

Excessive exposure to electronic media, including television, computers and handheld devices, during childhood has been associated with delayed language development, aggressive behaviour, smoking and obesity^(1–4)^. Recently, there has been increased interest in the potentially unique effects of media exposure during mealtime and its association with the development of obesity in children. Possible mechanisms to explain the link between mealtime media use and excess body weight include eating despite the lack of hunger, reduced satiety signals while watching media and exposure to advertisements promoting energy-dense foods and poor dietary habits^(5,6)^. A recent systematic review and meta-analysis including twenty observational studies (*n* 84 825) identified a positive association between television viewing during mealtime and risk of overweight/obesity in children^(7)^. While studies have focused primarily on television viewing during mealtime and its association with overweight and obesity, there is no evidence on the effects of media exposure during meals on other markers of cardiometabolic risk (CMR).

Non-HDL-cholesterol is an important marker of CMR in children and a significant predictor of dyslipidaemia in adulthood^(8,9)^. Lifestyle factors have been associated with circulating levels of non-HDL-cholesterol^(10–12)^, and patterns of non-HDL-cholesterol identified during early childhood have been shown to persist with age^(13)^. In addition to non-HDL-cholesterol, other CMR markers such as blood pressure, glucose and lipids have also been shown to persist into adulthood and are associated with increased risk of developing various diseases including the metabolic syndrome, type 2 diabetes and atherosclerotic plaques^(14,15)^. While systematic reviews assessing the association between television viewing/screen time and markers of CMR in children, including lipids, blood pressure, glucose and insulin, have yielded inconsistent results^(16–18)^, no studies to date have investigated the effects of mealtime media use on these CMR markers in preschool to school-aged children. The primary objective of the present study was to examine the association between total media use during mealtime and non-HDL-cholesterol levels as well as other CMR markers in children, including total cholesterol, HDL-cholesterol, TAG, glucose and insulin. Additionally, we examined whether media use during specific meals (i.e., breakfast, lunch, dinner and snacks) was associated with non-HDL-cholesterol and other CMR markers.

## Methods

### Study design and population

A repeated measures study was conducted in children ≥1 year of age participating in the TARGet Kids (The Applied Research Group for Kids) cohort, a primary care practice-based research network with ongoing recruitment described previously (ClinicalTrials.gov: NCT0186953)^(19)^. Healthy children younger than 6 years at first visit were recruited and followed during well-child visits at eleven paediatric or primary care centres in Toronto, Canada. Participants were invited to participate in annual data collection. Children with severe developmental delay, any chronic condition (with the exception of asthma) or whose families were unable to complete questionnaires in English were not included. The present study included children with recorded visits from January 2013 to July 2018 (*n* 6566). Children without non-HDL-cholesterol laboratory values due to lack of blood draw or assay failure (*n* 4141) and those without mealtime media use questionnaire responses (*n* 162) were excluded. Children who were <1 year of age for their only visit (*n* 144) were also excluded. Data collected prior to 2013 were not utilised as questionnaires asked only about television use during mealtime, and we were interested in all forms of electronic media use.

### Mealtime media use assessment

Parents were asked to complete a detailed Nutrition and Health Questionnaire at the following well-child visits: 12 months, 18 months, 2 years and yearly thereafter. The Nutrition and Health Questionnaire administered was modified from the Canadian Community Health Survey^(20)^ and contained questions on electronic media use during mealtime. For both a ‘typical weekday’ and ‘typical weekend day’, parents indicated typical electronic media usage during mealtime by responding to ‘Which meals did your child eat in a room with a screen device on (television, computer, tablet etc.)’, with Yes/No responses available for ‘Breakfast’, ‘Lunch’, ‘Dinner’ and ‘A snack’. The primary exposure variable of interest in the present study was total mealtime media use. This variable was created by adding all ‘Yes’ responses for each meal or snack and calculating a weighted average per week (i.e., multiplying weekday responses by 5 and weekend response by 2). Associations with total mealtime media use are reported in terms of both increasing meals per week and per day (total mealtime media use variable divided by 7). Electronic media use during individual meals (i.e., breakfast, lunch, dinner and snack) was calculated in the same way using only ‘Yes’ responses to ‘Breakfast’, ‘Lunch’, ‘Dinner’ and ‘A snack’. Associations with meal-specific media use are reported in terms of increasing meals per week.

### Outcome and covariate assessment

Non-fasting blood samples were collected by trained paediatric phlebotomists at the paediatric or primary care centres during yearly scheduled well-child visits. While blood samples were required for all participants at the time of initial recruitment, blood samples at follow-up visits were optional. Blood samples were then transported to Mount Sinai Services Laboratory, Toronto, Ontario for analysis as described previously^(21)^. Lipids, including total cholesterol, HDL-cholesterol and TAG, were measured using an enzymatic colorimetric assay. Non-HDL-cholesterol was calculated by subtracting HDL-cholesterol from total cholesterol. Glucose and insulin were measured using the enzymatic reference method with hexokinase and an electrochemiluminescence immunoassay, respectively. Generally, higher total cholesterol, non-HDL-cholesterol, TAG, glucose and insulin are associated with increased risk of developing various adverse health outcomes. Higher HDL-cholesterol is generally associated with a decreased risk of adverse health outcomes. Time of last meal/snack and non-water drink was recorded. Fasting time was accounted for in the current analysis, despite evidence indicating that fasting time may have little influence on serum lipid levels^(21,22)^. Children’s height and weight were measured by trained research assistants at clinical visits using a stadiometer (SECA) and precision digital scale (SECA model 703), respectively. Length boards were used for children under 2 years old. Age- and sex-standardised BMI *z*-scores (zBMI) were calculated using the WHO growth standards^(23,24)^.

Information on relevant covariates was assessed by parent-completed questionnaire during scheduled clinic visits. Covariates were identified *a priori* using the published literature and included child age^(25–31)^, sex^(25–35)^, birth weight^(32,36)^, total screen time^(27,34–36)^, maternal education^(29–32,34,36,37)^, maternal ethnicity^(25,27,31,35,38)^, family income^(25,27,33,36,38)^, parental history of cardiometabolic disease (high cholesterol, hypertension, heart disease and diabetes)^(39–41)^ and breast-feeding duration^(42)^. Additional covariates included unstructured free play time^(26,27,29,32,34,43)^, total screen time^(27,34–36)^ and family meals^(44)^. Unstructured free play was assessed using parents’ response to the open-ended question ‘Aside from time in daycare and school, on a typical weekday, how much time does your child spend outside in unstructured free play?’. Family meals were assessed by parents’ response to the open-ended question ‘In a typical week, how many times does your family eat the evening meal together?’. Finally, total screen time was assessed by calculating a weighted average of typical parent-reported weekday and weekend minutes spent awake in a room with the television on, videos or DVD on, playing on the computer, playing video games or playing with handheld devices^(45)^. Total screen time was included as a covariate so that results would likely be independent from overall media use and specific to media use during meals.

### Statistical analysis

Descriptive analyses of the primary exposure, outcomes and covariates were examined for all subjects at their first recorded visit (Table 1). For all analyses, repeated measures of exposures and outcomes measured concurrently over time were used to investigate the association between total mealtime electronic media use and outcome variables. Linear regression modelling using generalised estimating equations was used to account for within-subject correlation. Such models can accommodate subjects with available data at both single and multiple time points, and they do not require that all subjects have repeated measures. All subjects with at least one measure of total mealtime media use and non-HDL-cholesterol were included. A first-order autoregressive covariance matrix, AR(1), with observations ordered by subjects’ age in months, was used to account for correlations among repeated measures in all analyses^(46)^. The AR(1) covariance matrix accounts for correlation over time for a subject with repeated measures. Under this correlation structure, correlations decline with increasing time between visits.

**Table 1:** Baseline subject characteristics

Variable	Subjects (*n*)	Mean ± sd or *n* (%)
Participant characteristics		
Age (months)	2117	51·35 ± 31·68
Age groups	2117	
1–4 years		1282 (61)
5–13 years		835 (29)
Sex	2117	
Male		1138 (54)
Female		979 (46)
Maternal ethnicity	1853	
European		1181 (64)
East/Southeast Asian		188 (10)
South Asian		193 (10)
Other		291 (16)
Maternal education	2089	
High school, apprenticeship/trades certificate or no degree		187 (9)
College/university		1902 (91)
Self-reported household income	2061	
<$30 000		110 (5)
$30 000–$79 999		374 (18)
$80 000–$149 999		650 (32)
≥$150 000		927 (45)
zBMI (sd)	2108	0·15 ± 1·09
Birth weight (kg)	1881	3·25 ± 0·67
Average screen time (min/d)	1991	86·85 ± 76·11
Average weekday free play (min/d)	1988	50·56 ± 53·17
Maternal BMI	1691	25·15 ± 4·98
Parental cardiometabolic disease	1854	
Positive history		458 (25)
Negative history		1396 (75)
Family meals (evening meals with family/week)	2098	5·30 ± 1·93
Fasting time (h)	2037	2·04 ± 1·51
Exposure variables		
Total mealtime media use (meals/week)	2117	5·91 ± 7·17
Specific meal media use (meals/week)		
Breakfast	2117	1·63 ± 2·73
Lunch	2117	0·74 ± 1·93
Dinner	2117	1·20 ± 2·48
Snack	2117	2·34 ± 3·11
Outcome variables		
Non-HDL-cholesterol (mmo/l)	2117	2·67 ± 0·70
Total cholesterol (mmol/l)	2117	4·05 ± 0·73
HDL-cholesterol (mmol/l)	2117	1·38 ± 0·36
TAG (mmol/l)	2117	1·17 ± 0·66
Glucose (mmol/l)	2117	4·60 ± 0·68
Insulin (mmol/l)	2098	82·75 ± 70·00

In the primary analysis, the association between total mealtime electronic media use and CMR outcomes was investigated using both unadjusted and fully adjusted models. The fully adjusted model accounted for covariates that have been identified in the literature as potential confounders between mealtime media use and CMR outcomes or that have been directly associated with the outcome variables. Secondary analysis between mealtime media exposure during specific meals (breakfast, lunch, dinner and snack) and CMR outcomes was conducted using the same fully adjusted models. All analyses were stratified by *a priori* age groups (1–4 and 5–13 years). These age groups were selected since recent position statements on media use from both the American Academy of Pediatrics and the Canadian Paediatric Society specifically pertain to children ≥5 years^(47,48)^. Additionally, the majority of studies investigating mealtime television use and risk of overweight/obesity have been conducted in children aged 5 years and older^(7)^, and associations between mealtime media use and CMR markers may differ between these age groups. Interactions between mealtime media use and age group (1–4 years and 5–13 years) on each of the outcome variables were examined in models without stratification, but *P*-values and estimates of main effects are reported from models stratified by age groups, as this was specified *a priori*. For identified associations in either age group with *P* < 0·05, additional adjustment for zBMI was done as an exploratory analysis to determine whether the identified associations were likely independent from changes in zBMI.

In order to facilitate the inclusion of subjects with missing covariate data, multiple imputation (*m* = 20) was performed using the MICE package^(49)^ for all adjusted models. All covariates had missing observations <15 %, with the exception of maternal BMI (17 %). Participants with zBMI values > +5 or < −5 were excluded (*n* 2) in accordance with WHO guidelines^(50)^. The distributions of outcome variables were assessed, and both TAG and insulin were log-transformed in order to achieve normality. For all analyses, the *α*-error was set at 0·05 and statistical tests were two-sided. Statistical analysis was conducted using R version 3.5.1^(51)^.

## Results

A total of 2119 children ≥1 year of age had at least one TARGet Kids! visit between 2013 and 2018 with complete data on electronic media use during mealtime and blood lipids. After excluding zBMI outliers (*n* 2), the remaining 2117 subjects were included in the final analysis. Of the 2117 subjects with at least one recorded visit, 27 % (*n* 585) had repeated exposure and outcome data measured concurrently from two visits, and 10 % (*n* 209) had repeated measures from three or more visits, resulting in a total of 2911 observations. There were a total of 1619 (56 %) observations in the 1–4 years age group and 1292 (44 %) observations in the 5–13 years group. Baseline subject characteristics for all children included in the final analysis are shown in Table 1. Average total mealtime media use was 5·91 ± 7·17 meals/week among all children included in the present study at first visit. Participants had an average age of approximately 51 months and 54 % of children were male. The majority of mothers were of European descent (64 %) and had a college/university degree or higher (91 %).

Results of both unadjusted and fully adjusted models examining the association between total mealtime media use and CMR outcomes are shown in Table 2. Total mealtime media use was not associated with non-HDL-cholesterol in children 1–4 or 5–13 years old in both unadjusted (1–4 years, *P* = 0·42; 5–13 years, *P* = 0·15) and adjusted models (1–4 years, *P* = 0·10; 5–13 years, *P* = 0·29). In children aged 1–4 years, there was an inverse association between total mealtime media use and log-TAG in both unadjusted (*P* = 0·003) and fully adjusted models (*P* = 0·04). In the adjusted model, each increase of one meal with media *per day* was associated with a decrease in log-TAG (*β* = −0·03; 95 % CI −0·05, −0·0008). This is approximately equivalent to a 3 % decrease in TAG levels. The association between total mealtime media use *per day* and log-TAG years remained significant after additional adjustment for zBMI (*β* = −0·03; 95 % CI −0·05, −0·0003; *P* = 0·048). In children aged 5–13 years, total mealtime media use was inversely associated with HDL-cholesterol in both unadjusted (*P* = 0·03) and adjusted models (*P* = 0·003). In the adjusted model, each increase of one meal with media *per day* was associated with a decrease in HDL-cholesterol by −0·04 mmol/l (95 % CI −0·06, −0·01). After additional adjustment for zBMI, the association between total mealtime media use *per day* and HDL-cholesterol remained significant (*β* = −0·04; 95 % CI −0·06, −0·01; *P* = 0·004).

**Table 2:** Linear generalised estimating equations regression for the association between total mealtime media use per week and cardiometabolic outcomes stratified by age

	Unadjusted				Adjusted*			
	Estimate	95 % CI	*P*	*P*-int†	Estimate	95 % CI	*P*	*P*-int†
Non-HDL-cholesterol (mmol/l)								
Age 1–4 years	−0·002	−0·006, 0·003	0·43	0·10	−0·004	−0·01, 0·0008	0·10	0·06
Age 5–13 years	0·006	−0·002, 0·014	0·15		0·004	−0·004, 0·013	0·29	
Total cholesterol (mmol/l)								
Age 1–4 years	0·0002	−0·005, 0·005	0·95	0·68	−0·004	−0·010, 0·002	0·17	0·50
Age 5–13 years	0·002	−0·006, 0·01	0·62		−0·001	−0·010, 0·007	0·78	
HDL-cholesterol (mmol/l)								
Age 1–4 years	0·002	−0·0003, 0·004	0·09	0·005	0·0006	−0·002, 0·003	0·64	0·009
Age 5–13 years	−0·004	−0·007, −0·0004	0·03		−0·006	−0·009, −0·002	0·003	
Log-TAG (mmol/l)								
Age 1–4 years	−0·004	−0·008, −0·002	0·003	0·007	−0·004	−0·008, −0·00009	0·04	0·02
Age 5–13 years	0·003	−0·002, 0·008	0·17		0·003	−0·003, 0·009	0·29	
Glucose (mmol/l)								
Age 1–4 years	−0·004	−0·008, 0·001	0·12	0·59	−0·001	−0·007, 0·004	0·64	0·26
Age 5–13 years	−0·006	−0·01, 0·0004	0·07		−0·005	−0·012, 0·001	0·12	
Log insulin (mmol/l)								
Age 1–4 years	0·003	−0·003, 0·008	0·36	0·96	0·003	−0·003, 0·010	0·31	0·70
Age 5–13 years	0·003	−0·005, 0·01	0·45		0·0009	−0·0069, 0·0086	0·83	

* Adjusted for child age, child sex, birth weight, fasting time, unstructured free play, total screen time, maternal education, maternal ethnicity, family income, parental history of cardiometabolic-related disease, breast-feeding duration and family meals.

† *P*-values for *age* × *total mealtime media use* interactions estimated from unstratified models.

Results of the secondary analysis examining the association between media use during specific meals and non-HDL as well as other CMR markers were varied (shown in Table 3). There was no evidence for an association between media use during any specific meal and non-HDL-cholesterol in children aged 1–4 or 5–13 years (*P* > 0·05). In children aged 1–4 years, consuming an additional lunch with media *per week* was associated with decreased log-TAG (*β* = −0·01; 95 % CI −0·03, −0·002; *P* = 0·03). In children aged 5−13 years, consuming an additional breakfast with media *per week* was associated with decreased HDL-cholesterol (*β* = −0·012; 95 % CI −0·02, −0·004; *P* = 0·002), dinner with media was associated with increased log insulin (*β* = 0·02; 95 % CI 0·0007, 0·04; *P* = 0·04) and snacks with media were associated with decreased glucose (*β* = −0·02; 95 % CI −0·03, −0·003; *P* = 0·02).

**Table 3:** Linear generalised estimating equations regression for the association between media use during specific meals per week and cardiometabolic outcomes stratified by age*

	Breakfast				Lunch				Dinner				Snack			
	Adjusted estimate	95 % CI	*P*	*P*-int†	Adjusted estimate	95 % CI	*P*	*P*-int†	Adjusted estimate	95 % CI	*P*	*P*-int†	Adjusted estimate	95 % CI	*P*	*P*-int†
Non-HDL-cholesterol (mmol/l)																
Age 1–4 years	−0·008	−0·022, 0·005	0·22	0·14	−0·005	−0·02, 0·01	0·59	0·09	−0·01	−0·03, 0·002	0·10	0·19	−0·005	−0·017, 0·008	0·47	0·15
Age 5–13 years	0·008	−0·008, 0·024	0·34		0·03	−0·01, 0·07	0·16		0·002	−0·016, 0·019	0·86		0·004	−0·010, 0·017	0·60	
Total cholesterol (mmol/l)																
Age 1–4 years	−0·009	−0·023, 0·005	0·21	0·66	0·0006	−0·0177, 0·0190	0·94	0·28	−0·01	−0·03, 0·003	0·10	0·59	−0·003	−0·017, 0·010	0·65	0·56
Age 5–13 years	−0·004	−0·020, 0·012	0·60		0·02	−0·02, 0·06	0·28		−0·006	−0·024, 0·012	0·51		−0·002	−0·017, 0·013	0·76	
HDL-cholesterol (mmol/l)																
Age 1–4 years	−0·0007	−0·007, 0·006	0·84	0·03	0·006	−0·003, 0·014	0·20	0·09	−0·0004	−0·0071, 0·0063	0·90	0·13	0·002	−0·005, 0·008	0·62	0·10
Age 5–13 years	−0·012	−0·020, −0·004	0·002		−0·007	−0·021, 0·007	0·34		−0·008	−0·017, 0·002	0·11		−0·006	−0·013, 0·001	0·10	
Log-TAG (mmol/l)																
Age 1–4 years	−0·004	−0·014, 0·005	0·35	0·04	−0·01	−0·03, −0·002	0·03	0·29	−0·01	−0·02, 0·0004	0·06	0·39	−0·002	−0·012, 0·007	0·61	0·03
Age 5–13 years	0·010	−0·002, 0·021	0·12		−0·0006	−0·0258, 0·0245	0·96		−0·005	−0·019, 0·010	0·05		0·006	−0·003, 0·015	0·19	
Glucose (mmol/l)																
Age 1–4 years	−0·0002	−0·0131, 0·0127	0·98	0·93	0·004	−0·011, 0·019	0·60	0·25	−0·0007	−0·0151, 0·0137	0·92	0·63	−0·008	−0·021, 0·005	0·21	0·40
Age 5–13 years	0·002	−0·013, 0·017	0·80		−0·01	−0·04, 0·01	0·33		−0·005	−0·025, 0·014	0·60		−0·02	−0·03, −0·003	0·02	
Log insulin (mmol/l)																
Age 1–4 years	0·008	−0·007, 0·024	0·29	0·24	−0·0003	−0·0213, 0·0206	0·98	0·50	0·003	−0·014, 0·021	0·70	0·24	0·008	−0·007, 0·023	0·31	0·57
Age 5–13 years	−0·004	−0·022, 0·012	0·56		−0·01	−0·05, 0·02	0·44		0·02	0·0007, 0·04	0·04		−0·0007	−0·0161, 0·0147	0·93	

* Analyses adjusted for child age, child sex, birth weight, fasting time, unstructured free play, total screen time, maternal education, maternal ethnicity, family income, parental history of cardiometabolic-related disease, breast-feeding duration and family meals.

† *P*-values for *age* × *media use during specific meals per week* interactions estimated from unstratified models.

## Discussion

The current study provides some insight into the effects of mealtime media use on CMR markers independent of body weight. Our results suggest that total mealtime media use is likely not associated with circulating levels of non-HDL-cholesterol in pre-school (1–4 years) or school-aged children (5–13 years). In school-aged children, total mealtime media use was inversely associated with HDL-cholesterol, and this association was largely driven by media usage during breakfast. In pre-school-aged children, total mealtime media use was inversely associated with TAG, and there was evidence of an association between media use during lunch and decreased TAG in this age group.

Both the American Academy of Pediatrics and Canadian Paediatric Society recently released policy statements outlining the risks of weight gain with excessive media use in school-aged children and adolescents^(47,48)^. The Canadian Paediatric Society also specifically recommends that screen time be limited during family routines such as meals in pre-school-aged children^(52)^. In a US population of school-aged children, up to approximately 25 % of energetic intake was shown to be consumed while watching television. In our study population, children consumed an average of nearly six meals (21 % of total meals/snacks) with media per week. This may be of concern given both the positive association between mealtime media use and excess body weight^(7)^ and the high prevalence of obesity^(53)^. Furthermore, it is estimated that almost 40 % of children in Canada have abnormal values for at least one cardiovascular risk factor, including BMI, blood pressure, lipids and blood glucose^(54)^. Such unfavourable CMR profiles have been shown to track into adulthood and lead to an increased risk of developing metabolic syndrome, atherosclerosis and type 2 diabetes^(14,15)^.

Exposure to media during mealtime has several potentially unique effects which may make it an especially important risk factor in the development of increased CMR. Media consumption may act as a cue to stimulate eating in the absence of hunger^(5,6)^. Furthermore, media use during mealtime is thought to extend meal duration and ultimately increase energetic intake through reduced satiety signals^(6,55)^. Additionally, exposure to food advertisements on television encouraging the consumption of energy-dense foods during mealtime may promote poor dietary habits^(5,56,57)^. These factors likely all contribute to reported associations between mealtime media use and poor overall diet quality. Indeed, a recent systematic review identified associations between mealtime media use and lower scores on dietary quality indices. Specifically, watching television during mealtime was associated with decreased intake of fruits and vegetables, as well as an increased intake of fat- and sugar-containing foods including sugar-sweetened beverages^(58)^. Such dietary patterns have been shown to be associated with unfavourable CMR risk profiles^(59)^, suggesting that changes in diet quality associated with mealtime media may mediate the association with adverse CMR profiles in children.

We observed evidence of an inverse association between total mealtime media use and levels of HDL-cholesterol in children aged 5–13 years. This suggests that increased media use during mealtime may be associated with less favourable lipid profiles in school-aged children. Evidence of an inverse association between total mealtime media use and HDL-cholesterol remained after additional adjustment for zBMI, suggesting that the effects of mealtime media use on lipid profiles may be independent from effects on zBMI^(7)^. Understanding which specific meals are driving observed associations is important because it has the potential to inform future interventions and it gives parents a practical target to focus on. When examining specific meals, evidence of an inverse association with HDL-cholesterol was only observed for breakfast consumed with media. A cross-sectional study of 409 children aged 6–9 years in Iran identified a similar association between breakfast consumption while watching television and increased waist circumference as well as fasting blood sugars, but not lipids^(60)^. Other studies have shown that skipping breakfast is associated with unfavourable cardiometabolic profiles^(61)^, including decreased HDL-cholesterol in children^(62,63)^. It is therefore possible that media use during breakfast may act as a marker for irregular breakfast consumption patterns in the present study, possibly explaining its inverse association with HDL-cholesterol. Further research is necessary to determine the potential clinical significance of this association between mealtime media use and HDL-cholesterol.

The use of non-television forms of media such as smartphones and tablets was included in the current study. While the use of such devices among children has increased in recent years^(64,65)^, it is unclear whether interacting with these devices during meals has similar effects to watching television. A randomised crossover trial found that acute energy intake was lower in children who were using a computer while eating compared with children who were watching television^(66)^. Other studies conducted in adults^(67,68)^ also suggest that different forms of media may have varying effects on distraction from satiety signals and resulting energy intake. If the use of mobile media devices while eating results in less food consumption compared with watching television or no media at all, this could potentially explain the absence of associations identified between total mealtime media use and CMR markers in the present study. Such a phenomenon could also partially explain the unexpected inverse associations we observed between media use during snack time and glucose levels in children aged 5–13 years as well as media use during lunch and TAG in children aged 1–4 years. However, it must be noted that since our study was unable to differentiate between different types of media, no conclusions about possible differential effects of mobile media *v*. other types of media can be drawn. Further studies are necessary to determine whether mobile media use during mealtime has different effects from traditional television viewing on body weight and other CMR markers in children.

We observed some differences in the direction of associations between mealtime media use and CMR markers in our two *a priori* age groups. In children aged 5–13 years, total mealtime media use was inversely associated with HDL-cholesterol, suggesting that increased media use during mealtime may be associated with less favourable lipid profiles. Having more dinners with media was associated with increased insulin in this age group, although there was no evidence of an overall association between total mealtime media use (i.e., across all meals) and insulin. In children aged 1–4 years, we observed an unexpected inverse association between total mealtime media use and TAG. While TAG levels may be slightly affected by fasting time^(22)^, it is unlikely that fasting status confounded the observed association since it was adjusted for in the final analysis. The identified inverse association in this age group may also represent a spurious finding. Alternatively, it is possible that parents of pre-school-aged children in the present study utilised media during mealtime as a distraction tactic to increase food intake in picky eaters. Indeed, this motivation for television viewing during mealtime has been described by parents of 3−5-year-old children participating in a recent qualitative study^(69)^. This suggests that the effects of media use during mealtime could differ between pre-school and school-aged children. Further research examining the context in which mealtime media is used in children of different ages may help to better explain apparent differences in the direction of associations observed between age groups in the present study.

While our study has a number of strengths, including a large sample size of over 2000 children, the utilisation of repeated measures, the inclusion of non-television forms of media and adjustment for a number of relevant covariates, it does possess some notable limitations. Given the disproportionate representation of participants from households with relatively high income and maternal education, the results may not be generalisable to the broader Canadian population. Although the administered questionnaire included all types of media use during mealtime (i.e., television, computer, video game console and handheld devices), it does not ask about mobile media (i.e., handheld devices including tablets and mobile phones) and non-mobile media use separately. This precluded any analysis of differences between mobile and non-mobile media use during mealtime. Furthermore, parental estimates of media use during meals consumed away from home (e.g., at school) may not be accurate for school-aged children with mobile devices. Additionally, while the identified associations were statistically significant (*P* < 0·05), the effect sizes were relatively small. However, these small effects may be of importance if they occur chronically as cumulative exposure to lower levels of HDL-cholesterol from childhood through to adulthood has been associated with increased atherosclerosis^(70)^. Nevertheless, since these associations have not yet been replicated in independent cohorts, it is possible that any of the observed associations in the present study represent spurious findings. Finally, the observational nature of the study precludes the establishment of causality in any of the identified associations.

## Conclusion

While there was no evidence that mealtime media use was associated with non-HDL-cholesterol, it may be associated with unfavourable lipid profiles through effects on HDL-cholesterol independent of body weight in children ≥5 years. This suggests that promoting media-free meals in school-aged children may have beneficial effects on minimising CMR. Additionally, the motivation for mealtime media use among children and parents may differ between pre-school and school-aged children, which may contribute to the differing direction of associations observed in our study. Further studies which examine the context for mealtime media use may help further clarify results of the present study.
